# Fig latex inhibits the growth of pathogenic bacteria invading human diabetic wounds and accelerates wound closure in diabetic mice

**DOI:** 10.1038/s41598-022-26338-0

**Published:** 2022-12-17

**Authors:** Mohamed Salah, Gamal Badr, Helal F. Hetta, Walaa A. Khalifa, Ahmed A. Shoreit

**Affiliations:** 1 Botany and Microbiology Department, Faculty of Science, Assiut University, Assiut, 71516 Egypt; 2 Zoology Department, Faculty of Science, Assiut University, Assiut, 71516 Egypt; 3 Medical Microbiology and Immunology Department, Faculty of Medicine, Assiut University, Assiut, 71516 Egypt; 4 Department of Internal Medicine, Faculty of Medicine, Assiut University, Assiut, 71516 Egypt

**Keywords:** Cell biology, Immunology, Microbiology, Physiology

## Abstract

Impaired wound healing is one of the most critical complications associated with diabetes mellitus. Infections and foot ulcers are major causes of morbidity for diabetic patients. The current treatment of diabetic foot ulcers, commonly used antibiotics, is associated with the development of bacterial resistance. Hence, novel and more effective natural therapeutic antibacterial agents are urgently needed and should be developed against the pathogenic bacteria inhabiting diabetic wounds. Therefore, the current study aimed to investigate the impact of fig latex on pathogenic bacteria and its ability to promote the healing process of diabetic wounds. The pathogenic bacteria were isolated from patients with diabetic foot ulcers admitted to Assiut University Hospital. Fig latex was collected from trees in the Assiut region, and its chemical composition was analyzed using GC‒MS. The antibacterial efficacy of fig latex was assessed on the isolated bacteria. An in vivo study to investigate the effect of fig latex on diabetic wound healing was performed using three mouse groups: nondiabetic control mice, diabetic mice and diabetic mice treated with fig latex. The influence of fig latex on the expression levels of β-defensin-1, PECAM-1, CCL2 and ZO-1 and collagen formation was investigated. The GC‒MS analysis demonstrated the presence of triterpenoids, comprising more than 90% of the total latex content. Furthermore, using a streptozotocin-induced diabetic mouse model, topical treatment of diabetic wound tissues with fig latex was shown to accelerate and improve wound closure by increasing the expression levels of β-defensin-1, collagen, and PECAM-1 compared to untreated diabetic wounds. Additionally, fig latex decreased the expression levels of ZO-1 and CCL2.

## Introduction

A major health concern in the world today is diabetes mellitus (DM), which develops when the body is unable to produce or utilize insulin^1^. Globally, more than one in 10 adults are now living with diabetes. Moreover, there is a growing list of countries where one-in-five or even more of the adult population has diabetes^2^. According to the International diabetic federation (IDF) atlas the number of adults with diabetes is predicted to rise to 55 million by 2045^3^. The different complications of the uncontrolled DM include retinopathy, cardiovascular diseases, neuropathy, nephropathy, and diabetic wound (DW)^1,4^. Patients with type 2 diabetes are more likely to suffer from these wounds, which are positively correlated with disease duration and the burden of complications^5^. Patients with uncontrolled diabetes have a high risk for diabetic foot ulcers (DFUs)^6^. Globally, it is estimated that 6.3% of diabetic patients have foot disease^7^, with North America generally having a higher regional incidence, which has been reported to reach 13% in some population studies^8,9^. It is anticipated that between 19 and 34% of people with diabetes will have a DFUs at some point in their lives^6,10^. Additionally, patients with DFUs are 2.5 times more likely to die than diabetic patients without foot disease; therefore, DFUs lead to significant mortality for affected patients^11^. DFUs affect 42.2% of diabetics and impose an economic burden on the health sector, that bears a cost of 3 billion dollars for the treatment every year^12^. Diabetic foot disease is one of the most challenging complications of diabetes to treat, and it has become a major cause of nontraumatic amputation^13^. In every 30 s, one person with diabetes is known to undergo lower extremity amputation somewhere in the world^14^. During diabetes, delayed wound healing is a result of different factors, including bacterial infections^15^. Indeed, 80% of patients with DFUs infected with pathogenic biofilm-forming bacteria need lower-limb amputation^16^. Hospitalization, amputation, and length of care have all been identified as major contributors to high health-care costs in the United States and elsewhere^17,18^. Treatments that promote speedy and complete healing of DFUs minimize the need for hospitalization, decrease the probability of amputation, and limit health-care costs^19^. DFUs complicated by diabetic foot infections (DFIs) are extremely probable^20^. The amputation rate in DFU patients is 38.4%^21^. Infection is a common (> 50%) side effect of DFUs^22–24^. New evidence emphasizes the significance of biofilm infection in the progression of nonhealing DFUs^25^. DFIs are polymicrobial in nature, and the growth mode of these microbes is complicated by the biofilm formation mechanism^26,27^. The inflammatory phase, which begins on the first day of wound injury and lasts for 10 days for wound repair, is a crucial stage in which colonizing pathogenic bacteria are encountered during tissue repair^28^. Most bacteria are genetically resistant to many antibacterial drugs. Because of the high morbidity rate, a large number of patients in hospitals are exposed to new infections due to the spread of pathogenic bacteria and drug resistance^29,30^. Therefore, multidrug-resistant bacteria contribute to delaying the steps of the healing process and result in the formation of chronic wounds^28^. Gangrene is one of the major and most severe consequences of bacterial infection associated with diabetes and is due to hyperglycemia leading to the failure of the immune response to control the spread of invading pathogens^31,32^. Polymicrobial biofilm aggregates have emerged as a major danger in the broader discipline of wound infection, as it is in this form that microbes acquire more resistance to host defenses and antibiotics, in addition to acquiring pathogenicity^33,34^. Pathogenic and commensal bacteria coaggregate synergistically in a pathogenic biofilm to establish a persistent infection^35^. Early identification and treatment of pathogenic microorganisms remain critical therapeutic goals that must be achieved^36^. Pathogenic bacteria such as *Staphylococcus* sp., *Pseudomonas* sp. and *Bacillus* species have been found to play a major role in attachment to catheters due to their ability to form biofilms^37^. Biofilms act as a mechanical barrier to antimicrobials and immune system cells and contribute to multidrug resistance^38^. Although *Staphylococcus haemolyticus* is considered a normal microbiota of the skin and an opportunistic pathogen associated with hospital-acquired infections, infections caused by it are increasing. Regardless of its low virulence profile, *S. haemolyticus* poses a serious risk to patients with DFUs due to its multidrug resistance^39^. Another concern with *Staphylococcus* infections is that some bacteria can produce biofilms to evade host immune systems and protect themselves against therapies, which play a crucial role in the virulence and pathogenicity of *Staphylococcus* spp. Nonhealing DFUs are distinguished by impaired wound healing associated with recurrent *Staphylococcus aureus* infection and unresolved inflammation^40^. A previous study tested *S. haemolyticus* strains isolated from bloodstream infections and discovered that 34% of them could form biofilms in vitro^41^. Moreover, multidrug-resistant *Pseudomonas aeruginosa* infection has become a challenge in clinical practice^42–44^. In the treatment of DFUs, commonly used antibiotics are linked to negative side effects, such as irritation, hyperpigmentation, histocompatibility, tissue rejection and the emergence of bacterial resistance^45,46^. Thus, empiric treatment for infected DFUs may fail if there is microbial resistance^47^. Moreover, the choice of the appropriate antibiotics for the infection is difficult; furthermore, coselection may involve resistance genes^48^. Globally, antibiotic resistance is a significant threat. According to the World Health Organization (WHO), “The world urgently needs to change the way it prescribes and uses antibiotics.”^49^. Therefore, this problem can be solved by developing new medications with minimal or no side effects and without developing new resistant microbes. Consequently, the usage of natural compounds in pharmaceuticals has greatly expanded. According to one of the World Health Organization (WHO) performed investigations, approximately 80% of the global population received medications containing chemicals produced from natural compounds^50–53^ for a wide range of infections. Many phytochemicals are antibacterial and play an integral role in healing therapies^54^. The biomolecular compounds generated and extracted from plants are a valuable source of antibacterial and anti-biofilm-forming pathogenic bacteria^55^. Plants have been used for therapeutic purposes because they are associated with fewer toxic effects and are more economical than manufactured therapies^56,57^.

The fluid in some laticiferous plants called latex contains bioactive compounds with valuable antibacterial and antioxidant effects. Latex is a biological fluid secreted by a wide range of plants and is composed of several types of metabolites, such as polyisoprene and sugars. The protein compounds of latex (peptidases, lipases, chitinases, steroids and numerous secondary metabolites) are commonly found in various latex fluids. In addition to the industrial importance of plant latex as a rubber source, as from the para rubber tree (*Hevea brasiliensis*), latex is a component of plant defense against microbes^58^. The stored latex in specialized cells called laticifers is secreted in response to any plant physical damage or wounding^58^.

In contrast to most latex-bearing plants, which store small, limited amounts of latex, the fruit of the fig tree (*Ficus carica*) exudes a high amount of latex. From ancient times to the present, fig latex has been used in India for people who suffer cracks in the mouth or on the lips or tongue because it is an excellent tonic. This effect may be due to fig latex enzymes such as ficin, proteases, lipodiastase, and amylase^59^. Plant latex is frequently applied topically to a variety of wounds and plays a key role in wound care^60–62^. Many endogenous proteases are involved in the various stages of wound healing during the physiological healing process; similarly, latex proteases used to treat wounds will act on various stages of wound healing^63^. For instance, procoagulant and thrombin-like proteases function to restore hemostasis during the early stages of wound healing. In the later stages of wound healing for debridement, plasmin-like and other ECM-degrading proteases assist, and some mitogenic proteases aid in cell proliferation and angiogenesis. Plant latex collagenolytic and other ECM-degrading proteases could play a role in the remodeling of collagen and other extracellular matrix (ECM) components^62^.

In addition to the increase in health complications while using chemically synthetic drugs, the large-scale use of antibiotics leads to different strains of multidrug-resistant bacteria. The latex of figs has been applied on a large scale in the treatment of warts, skin ulcers and sores and can be taken as a purgative and vermifuge^59^. Fig latex is useful for milk coagulation and can be used as a lipid-lowering drug due to the presence of triterpenoids^64^. Fig latex is considered a restorative natural material that helps in quick recovery after prolonged illness^65^. Due to its antimicrobial activities, low toxicity, and availability, fig latex represents a remedy for several health complications^66^. Nevertheless, no published work has demonstrated the antibacterial activities of fig latex against the pathogenic bacteria inhabiting diabetic wounds. Thus, the current study is a pioneer study evaluating the potential impact of fig latex on pathogenic bacteria isolated from human DFUs in vitro. Furthermore, the study aimed to clarify the efficacy of fig latex in accelerating the healing process of diabetic wound skin tissues in a streptozotocin (STZ)-induced diabetic mouse model in vivo by monitoring the expression of some proteins related to the healing process, such as β-defensin (antibacterial protein) and zonula occluden-1 (ZO-1), using ELISA. Additionally, the expression levels of CCL2 and PECAM-1 were detected using immunohistochemistry (IHC).

## Results

### Gas chromatography‑mass spectrometry (GC–MS) analysis of fig latex

**Figure 1:**
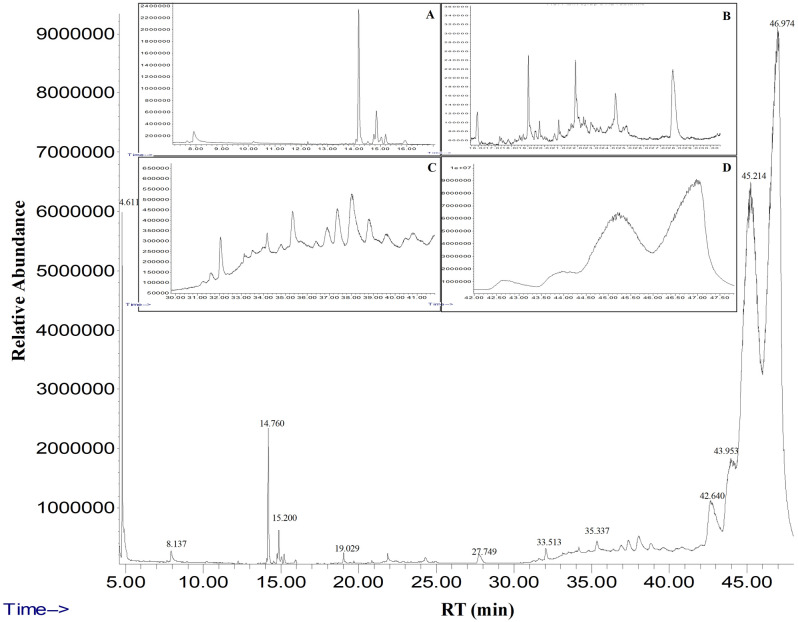
Gas chromatography and mass spectrometry chromatogram of fig latex analysis. The analysis demonstrated several peaks with different retention times to 47 min of twenty-four active constituents. A, B, C and D are zoomed regions of the chromatogram, RT, retention time.

The GC‒MS analysis of fresh fig crude latex showed the presence of twenty-four bioactive compounds with concentrations more than 0.1% of the total latex contents. The most abundant compound with the highest percentage was taraxasterol acetate (more than 50% of the total latex content). Moreover, the olean-12-en-3-ol, acetate, (3.beta.)- was detected and represented 32% of the total latex content. Finally, 9,19-cyclolanost-24-en-3-ol, (3.beta.)- (3.999%) and Lanosta-8,24-dien-3-ol, acetate, (3.beta.)- (3.346%) were detected. All four major chemical constituents represented 90% of the latex. Other active compounds, along with their concentration (peak area %) and retention time (RT), are presented with the chromatogram in Fig. 1, and the chemical components obtained from the GC‒MS analysis are presented in detail in Table 1.

**Table 1:** Chemical composition of *Ficus carica* latex detected by GC‒MS.

S. No	RT (min)	Compound	Area (%)	M. formula	M. wt (g/mol)	CAS registry No
1	4.611	2-Hexanone	0.156	C_6_H_12_O	100.16	591-78-6
2	6.137	3,3-Dimethyl-5-oxohexanoic acid	0.619	C_8_H_14_O_3_	158.19	n/a
3	14.081	α-Gurjunene	0.242	C_15_H_24_	204.35	489-40-7
4	14.760	Humulene	1.305	C_15_H_24_	204.357	6753-98-6
5	15.038	Nonadecane	0.375	C_19_H_40_	268.518	629-92-5
6	15.200	GERMACRENE-D	0.104	C_15_H_24_	204.35	23986-74-5
7	15.944	γ-Elemene	0.102	C_15_H_24_	204.35	20307-84-0
8	19.029	Pentadecane	0.1	C_15_H_32_	212.421	629-62-9
9	27.749	1-[5-(2-Methylphenoxy)pentyl]piperidine	0.119	C_17_H_27_NO	261.403	5315-11-7
10	32.057	cis-13-Eicosenoic acid	0.400	C_20_H_38_O_2_	310.51	17735-94-3
11	33.124	Silane,[[(3.beta.)-lanosta-8,24-dien-3-yl]oxy]trimethyl-	0.235	C_33_H_58_OSi	498.9	87649-55-6
12	33.513	Oleic acid	0.100	C_18_H_34_O_2_	282.461	112-80-1
13	35.337	trans-Geranylgeraniol	0.102	C_20_H_34_O	290.483	7614-21-3
14	36.915	DDT	0.270	C_14_H_9_Cl_5_	354.486	50-29-3
15	37.374	2,6,10,14,18,22-Tetracosahexaene,2,6,10,15,19,23-hexamethyl-, (all-E)-	0.215	C_30_H_50_	410.718	21681-17-4
16	38.008	2,5-di-tert-Butyl-p-quinone	0.405	C_14_H_20_O_2_	220.307	2460-77-7
17	38.810	9-(4-Aminoanilino)-7-methylimidezo[4,5-f]quinolone	0.752	C_12_H_11_N_3_	197.2	n/a
18	39.658	cis-Vaccenic acid	0.205	C_18_H_34_O_2_	282.461	506-17-2
19	40.473	trans-δ (sub 9)-Octadecenoic acid	0.207	C_18_H_34_O_2_	282.5	5684-82-2
20	40.809	9,19-Cycloergost-24(28)-en-3-ol,4,14-dimethyl-, (3.beta.,4.alpha.,5.alpha.)-	0.251	C_32_H_52_O_2_	468.754	10376-42-8
21	42.640	Lanosta-8,24-dien-3ol, acetate, (3.beta.)-	3.346	C_32_H_52_O_2_	468.754	2671-68-3
22	43.953	9,19-Cyclolanost-24-en-3-ol, (3.beta.)-	3.999	C_30_H_50_O	426.7	469-38-5
23	45.214	Olean-12-en-3-ol, acetate, (3.beta.)-	32.094	C_32_H_52_O_2_	468.8	1616-93-9
24	46.974	Taraxasterol-acetate	51.359	C_32_H_52_O_2_	468. 754	6426-43-3

The data included in the table clarify the range of twenty-four different bioactive chemical constituents at different concentrations ≥ 0.1 of the total latex content obtained within different retention times. RT, retention time per minute; Compound, active compounds detected by GC‒MS; (%), percentage of compound; M. formula, molecular formula; CAS registry No, number assigned to a substance when it enters the CAS REGISTRY database; M. wt, molecular weight of the compound.

### Fig latex exerts antibacterial activity using the well diffusion method

**Figure 2:**
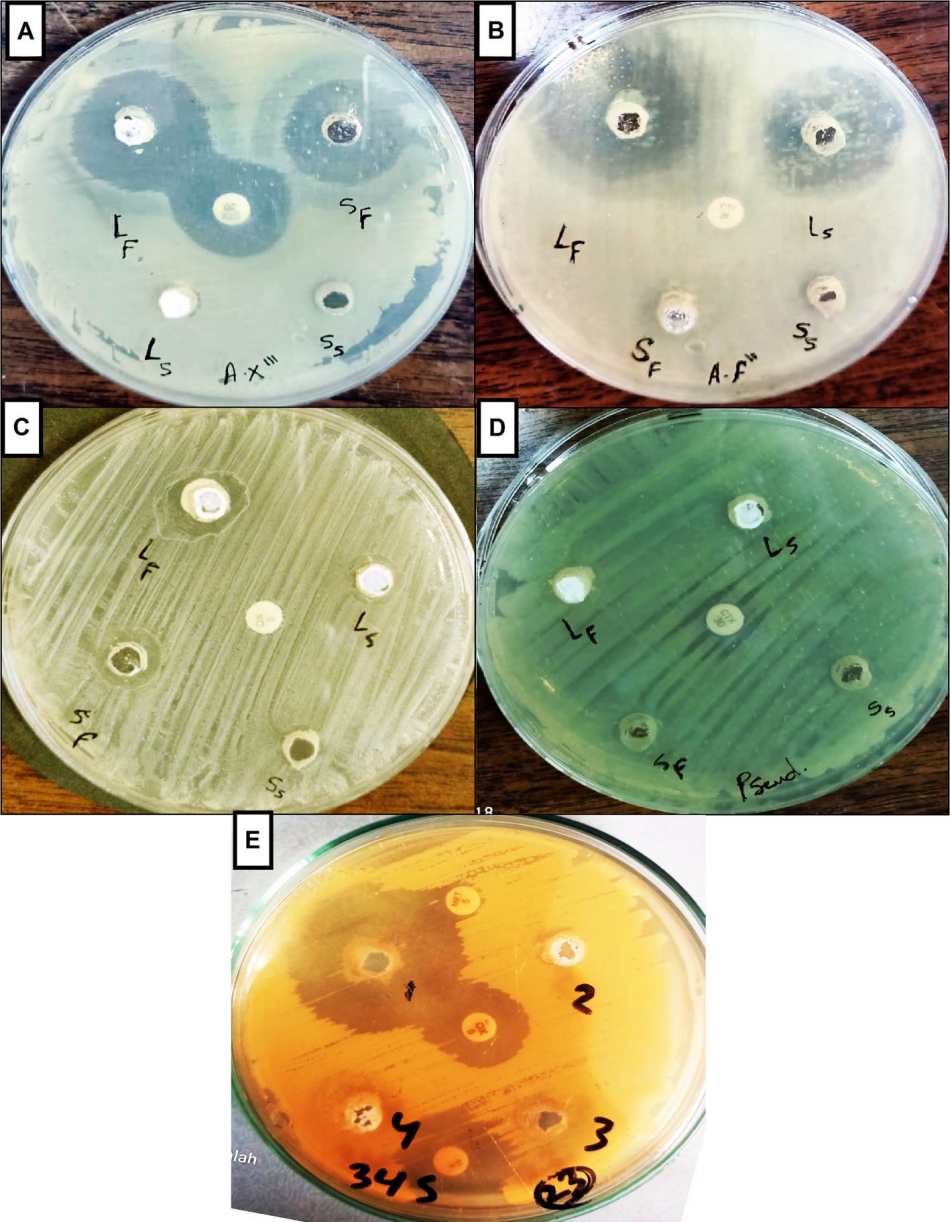
Inhibition zone showing the antibacterial activity of fresh fig latex on bacteria isolated from diabetic wounds. Causing a clear zone with diameter against the pathogenic bacteria (**A**) *Paenibacillus* sp., (**B**) *Bacillus* sp., (**C**) *Pseudomonas* sp. MS2, (**D**) *Pseudomonas* sp. *MS4* and (**E**) *Staphylococcus haemolyticus* AUMC b-331*.*

After the bacteria were obtained from DFUs using a swab method, the bacterial community was screened. We isolated many pure strains using the streak plate method, spreading bacteria in different culture media, such as MSA for gram-positive bacteria and MacConkey agar for gram-negative bacteria. The antibacterial activity of fresh fig latex was tested against five DFU biofilm-forming bacterial isolates*, Staphylococcus haemolyticus* AUMC B-331, two Gram-negative *Pseudomonas* sp*.*, *Bacillus* sp., and *Paenibacillus* sp., to determine the efficacy of the latex as an antibacterial drug. The diameter of the inhibition zone is shown in Fig. 2. The largest clear zones formed by fresh fig latex were 32, 30, and 28 mm, against *St. haemolyticus AUMC B-331*, *Bacillus* sp., and *Paenibacillus* sp., respectively, whereas the smallest clear zone observed was 18 mm against *Pseudomonas* sp. MS2, and there was no significant effect with no clear zone or growth inhibition against *Pseudomonas* sp. MS4. Stored latex showed less activity than fresh crude latex, as presented in Table 2.

**Table 2:** Inhibition zone of antibacterial activity of fig latex against biofilm-forming bacteria isolated from DFUs.

Inhibition zone diameter^a^ (mm)						
Strain	Fresh latex	Stored latex	Oxacillin (5 μg/disc)	Methicillin 5 mcg	Tetracycline (30 μg/disc)	Cefotaxime ctx 30 mcg
*Paenibacillus* sp. (A)	28	9	–	–	–	22
*Bacillus* sp. (B)	30	26	–	–	–	10
*Ps. MS2* (C)	18	12	–	–	–	–
Ps. MS4 (D)	–	–	–	–	–	14
*Staphylococcus haemolyticus* AUMC B-331 (E)	32	10	20	10	25	–

Fresh, fresh fig latex activity; stored, stored fig latex activity. Oxacillin, methicillin, tetracycline and cefotaxime are the antibiotics used as standard references, no activity; ^a^Inhibition zone includes the well diameter.

### Determination of the minimum inhibitory concentration (MIC) and minimum bactericidal concentration (MBC)

**Figure 3:**
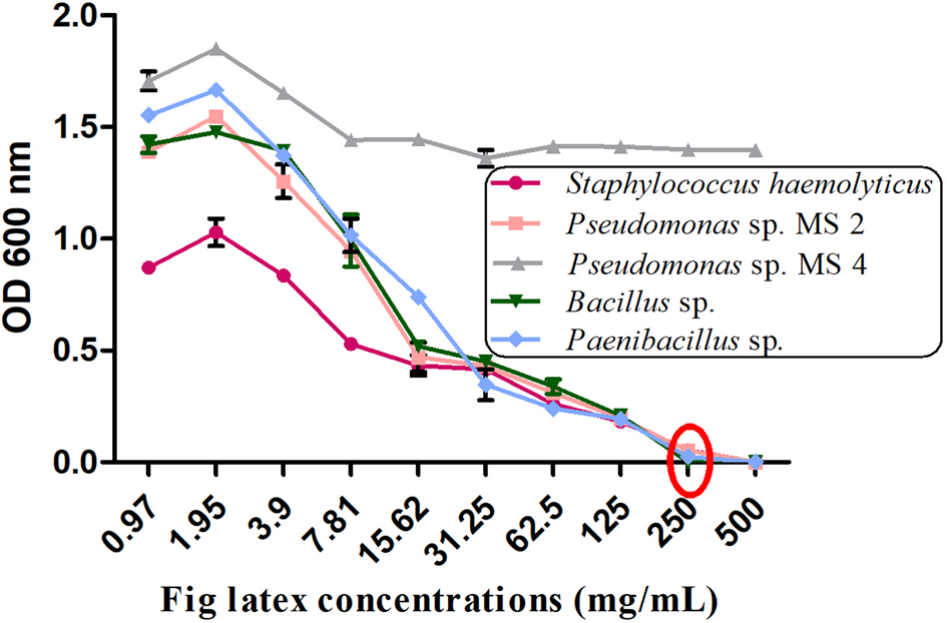
Minimum inhibition concentration (MIC) of fig latex for gram-positive and gram-negative bacteria. MIC reduction is presented as the decrease in the OD600 of the bacterial suspension treated with latex at various concentrations (0.97, 1.95, 3.9, 7.81, 15.62, 31.25, 62.5, 125, 250 and 500 μg/ml) against *St. haemolyticus, Ps. MS2, Ps. MS4, Bacillus* sp. *and Paenibacillus* sp. showing the MIC at 250 mg/ml with a red circle.

The MIC value was found to be 250 mg/mL, demonstrating a promising effect of fresh fig latex as a natural antibacterial agent. According to broth macrodilution results, the MIC was 250 mg/ml for *St. haemolyticus AUMC B-331, Bacillus* sp., *Pseudomonas* sp. *MS2,* and *Paenibacillus* sp., and the MBC was 500 mg/mL; the MIC of fig latex for *Pseudomonas* sp. MS4 was not reported (Fig. 3).

### Fig latex reduces bacterial biofilm formation

**Figure 4:**
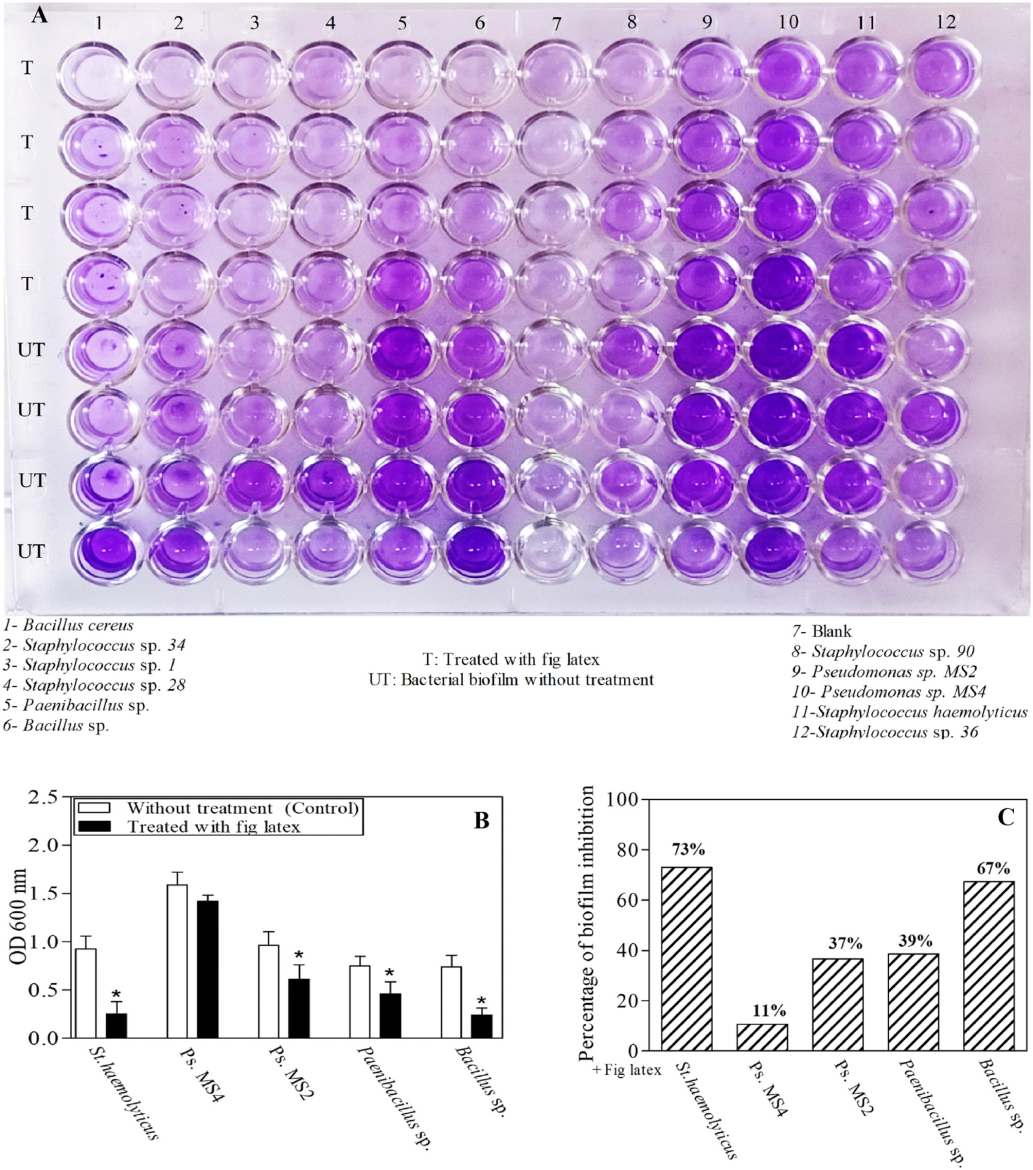
The impact of fig latex on bacterial biofilm using a microtiter plate biofilm assay against five pathogenic bacterial isolates from diabetic foot ulcers. (**A**) Microtiter plate showing the differences in the color intensity; (**B**) Quantitative determination of the optical density of the biofilm from the isolated bacteria as control (open bars) and after treatment with latex (closed bars); (**C**) Percentage of the antibiofilm activity of fig latex against the bacterial isolates (*St. haemolyticus, Ps. MS2, Ps. MS4, Bacillus* sp. and *Paenibacillus* sp*.*)*.*

The results of the plate shown in Fig. 4A were quantified using a microplate reader at OD 600 nm, and it was revealed that there were five isolates forming the biofilm: *Staphylococcus haemolyticus AUMC B-331, Pseudomonas* sp. *MS2, Pseudomonas* sp. *MS4, Bacillus* sp., and *Paenibacillus* sp. Low, moderate, and high bacterial biofilm-forming ability was interpreted when OD600 nm was < 1, 1–2.9 and > 2.9, respectively. Accordingly, *Pseudomonas* sp. MS4 showed high biofilm-forming ability, while *Pseudomonas* sp. MS2 and *St. haemolyticus* showed moderate ability. On the other hand, *Bacillus* sp. and *Paenibacillus* sp. had low biofilm-forming ability. In Fig. 4B, the ability of fig latex to reduce the bacterial biofilm formed by these isolates is illustrated. Notably, it was found that fresh fig latex at the MIC had the ability to significantly reduce the biofilm formed by four isolates out of the five tested, *Staphylococcus haemolyticus AUMC B-331, Pseudomonas* sp. *MS2, Bacillus* sp., and *Paenibacillus* sp. However, it had no significant activity against *Pseudomonas* MS4 sp. The percent biofilm inhibition after treatment with fig latex is shown in Fig. 4C. Additionally, after treatment with fig latex, the biofilm formed by *Staphylococcus haemolyticus* AUMC B-331 was decreased by 73%*,* that formed by *Pseudomonas* sp. MS2 was decreased by 37%, that formed by *Paenibacillus* sp. was decreased by 39% and that formed by *Bacillus* sp. was decreased by 67%. Additionally, fig latex inhibited the biofilm formed by *Pseudomonas* sp. MS4 by only 11%, showing that there was no significant effect of latex on *Pseudomonas* sp. MS4.

### Survival curve of DFU pathogenic bacteria in the presence of fig latex

**Figure 5:**
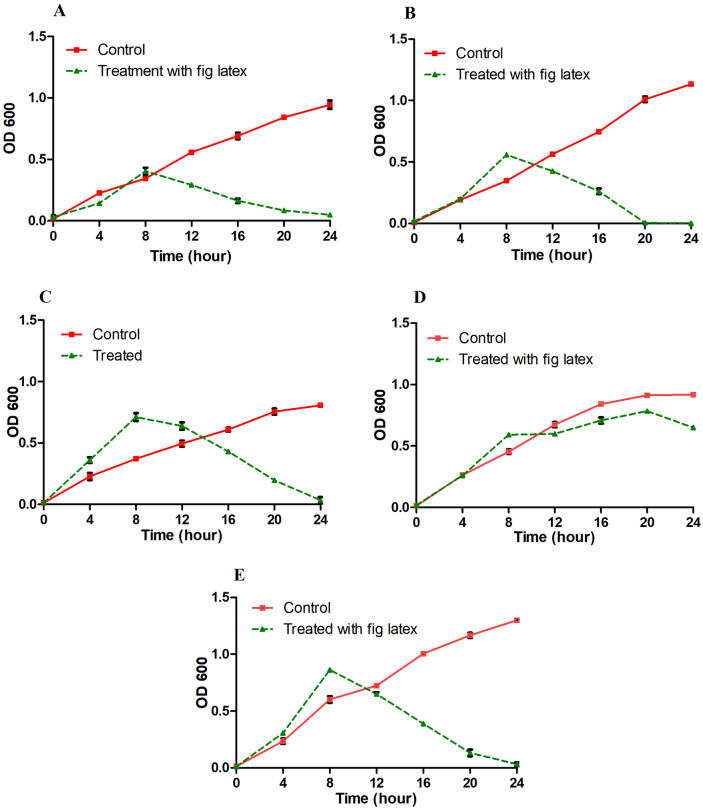
Survival curve of DFU pathogenic bacteria in the presence of fresh fig crude latex. The curves represent the bacterial isolates supplemented with the MIC of fresh fig latex in TSB media (dashed line) and show a significant decrease in OD600 for (**A**) *Paenibacillus* sp., (**B**) *Bacillus* sp., (**C**) *Pseudomonas* sp. MS2, and (**E**) *Staphylococcus haemolyticus* AUMC B-331 but not for (**D**) *Pseudomonas* sp. MS4. The curves represent the bacterial isolates in TSB media without latex (solid line) and show an increase in OD at 600 nm. The results are the mean from triplicate independent measurements.

The survival curve test of isolated bacteria demonstrated a slightly similar increase in growth over 24 h. The growth curves were decreased in the case of the treatment of the bacterial culture with the MIC of fresh fig latex after nearly 8 h, with a significant growth inhibition starting at 16 h of culture with fig latex, as demonstrated in Fig. 5A–C, E. In contrast, the growth curve of *Pseudomonas* sp. MS4 did not show any significant decrease after the treatment of the culture with fig latex, suggesting that it is a strain resistant to fig latex (Fig. 5D).

### Fig latex enhances the healing process of diabetic wounds

**Figure 6:**
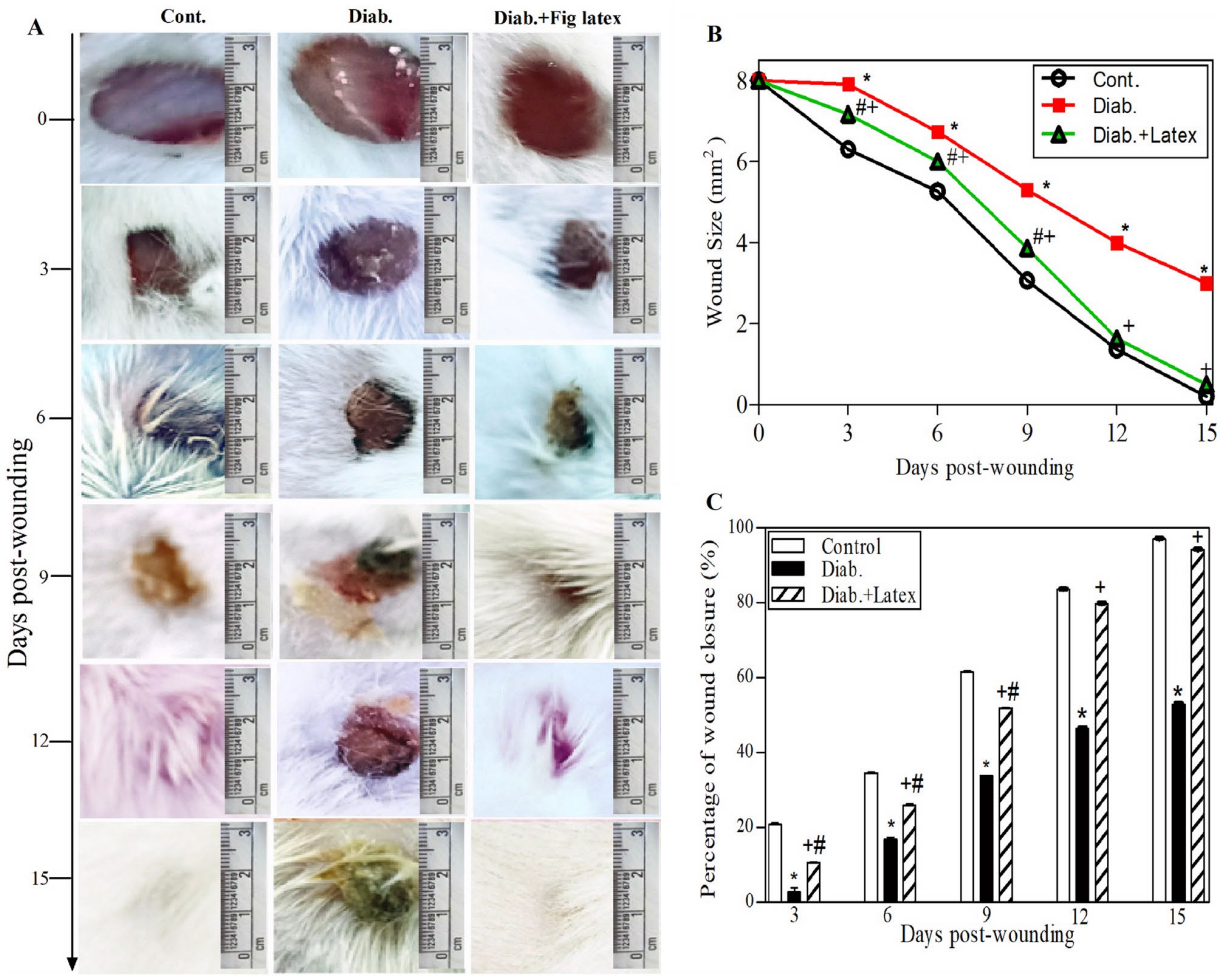
Effect of fig latex on wound healing and wound closure in diabetic mice. (**A**) Photographs showing the effect of fig latex on accelerating the healing process in diabetic wounds at each time point from Day 0 (the day of wound creation) until Day 15 (final point). (**B**) The accumulated data from three individual mice in each group at each time point post-wounding represent the change in diameter (mm). (**C**) The accumulated results from three animals in each group for each time point post-wounding were calculated by the previously mentioned equation in the Methods section, and the results show the percentage of wound closure. The data are expressed as the mean ± SEM. *P < 0.05, Diab. versus cont., ^+^*P* < 0.05, diabetic mice treated with Latex versus cont., ^#^*P* < 0.05, diabetic treated with Latex versus diabetic group.

The in vivo experiment on the mouse model, shown in Fig. 6A, monitored the morphological changes occurring in the three animal groups at each time point from Day 0 to Day 15 post-wounding. It was noted that in the diabetic animals, the healing process of wounds was delayed, and abscesses were found in the diabetic wounds with no healing compared to the healing process of wounds in the control group. Most importantly, topical application of fig latex on the diabetic wounds twice daily improved and accelerated the wound closure near that found in the control group. Figure 6B shows the wound size starting from 8 mm in the three animal groups at Day 0 post-wounding. The diabetic group showed a significant increase in the wound diameter (4 mm) (impaired healing) compared to the wound diameter (0 mm) in the control group at Day 15 post-wounding. Interestingly, when the wounds were topically treated with fresh fig latex, the wound diameters (0 mm) were similar to those found in the control group. The percentage of wound closure was calculated from the accumulated data (Fig. 6C). The diabetic animals exhibited a significant decrease in the percentage of wound closure compared to the control nondiabetic group. Amazingly, when the diabetic wounds were topically treated with fresh fig latex, the percentage of wound closure was significantly enhanced a level similar to that found in the control group.

### Fig latex restores collagen formation in the wounded skin tissues of diabetic mice

**Figure 7:**
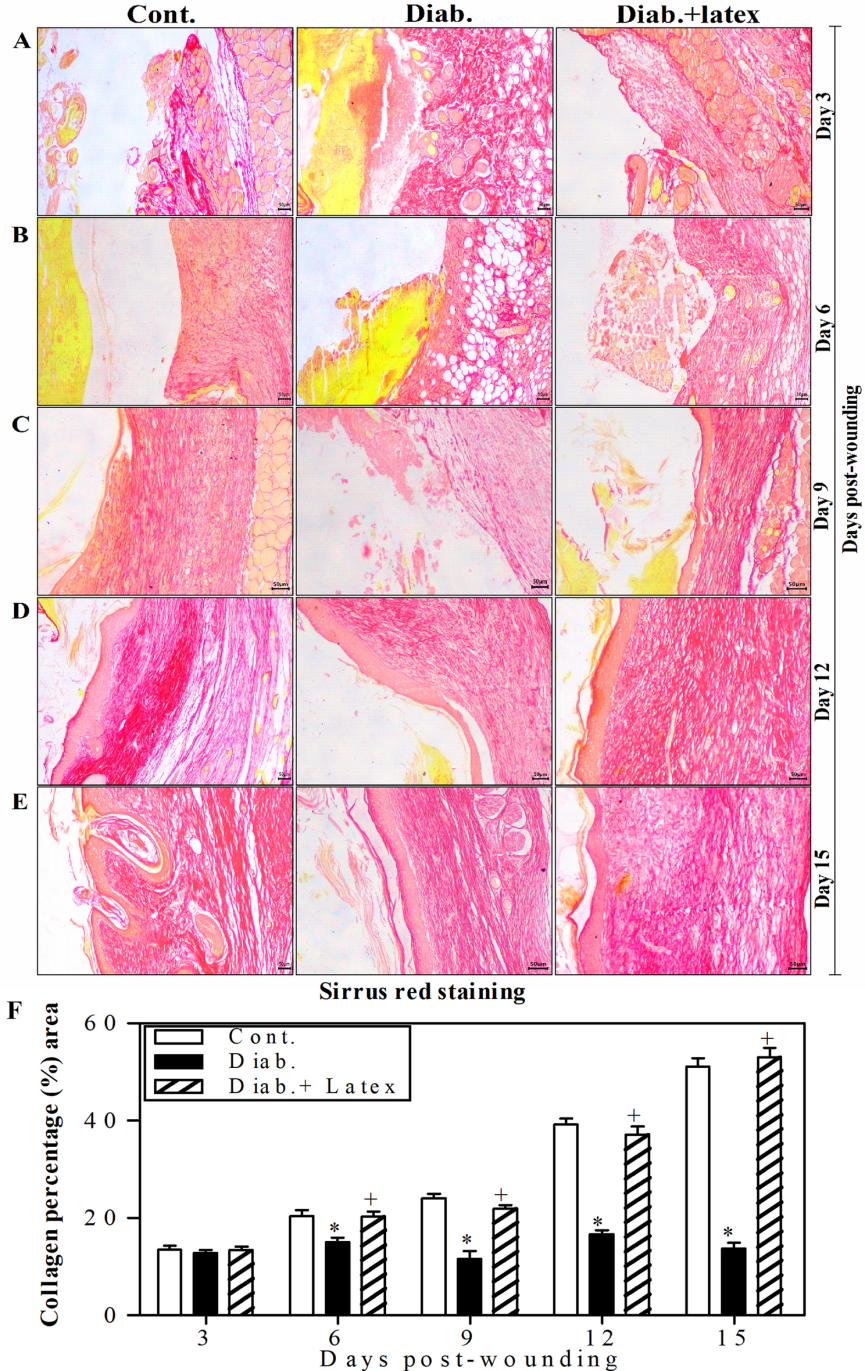
Collagen deposition in the wounded skin tissues stained with Sirius red. Collagen formation (red color) was detected in the wounded skin tissues of the three animal groups using Sirius red staining. One representative experiment is shown at 3 (**A**), 6 (**B**), 9 (**C**), 12 (**D**) and 15 days (**E**) post-wounding. (**F**) The percentages of collagen formation were quantified in the three animal groups, and the accumulated data from three animals from each group are expressed as the mean percentage of collagen formation ± SEM in control mice (open bars), diabetic mice (closed black bars) and diabetic mice treated with fig latex (hatched bars). *P < 0.05, diabetic versus control; and ^+^*P* < 0.05, diabetic-treated with fig latex versus diabetic animals.

Figure 7A–E shows representative pictures of wounded skin tissues stained with Sirius red to monitor collagen deposition. In Row A, at Day 3, the three animal groups showed similar collagen deposition. However, from Row B to E, it was found that from Day 6, the expression of collagen began to obviously increase in the control group over time until reaching the maximal expression at Day 15 post-wounding. However, the diabetic animals showed an obvious decrease in the expression of collagen compared to the control animals. Most importantly, the treatment of diabetic mice with fig latex restored collagen expression to levels similar to those found in the control group. Using ImageJ software, the collagen expression in three animals per group was quantified, and accumulated data are presented in Fig. 7F. From Day 6 to Day 15 post-wounding, both control and fig latex-treated diabetic animals demonstrated similar expression of collagen in wounded skin tissues. Nevertheless, the diabetic animals exhibited a significant decrease in the expression of collagen.

### Fig latex enhances vascularization in wounded diabetic skin tissues

**Figure 8:**
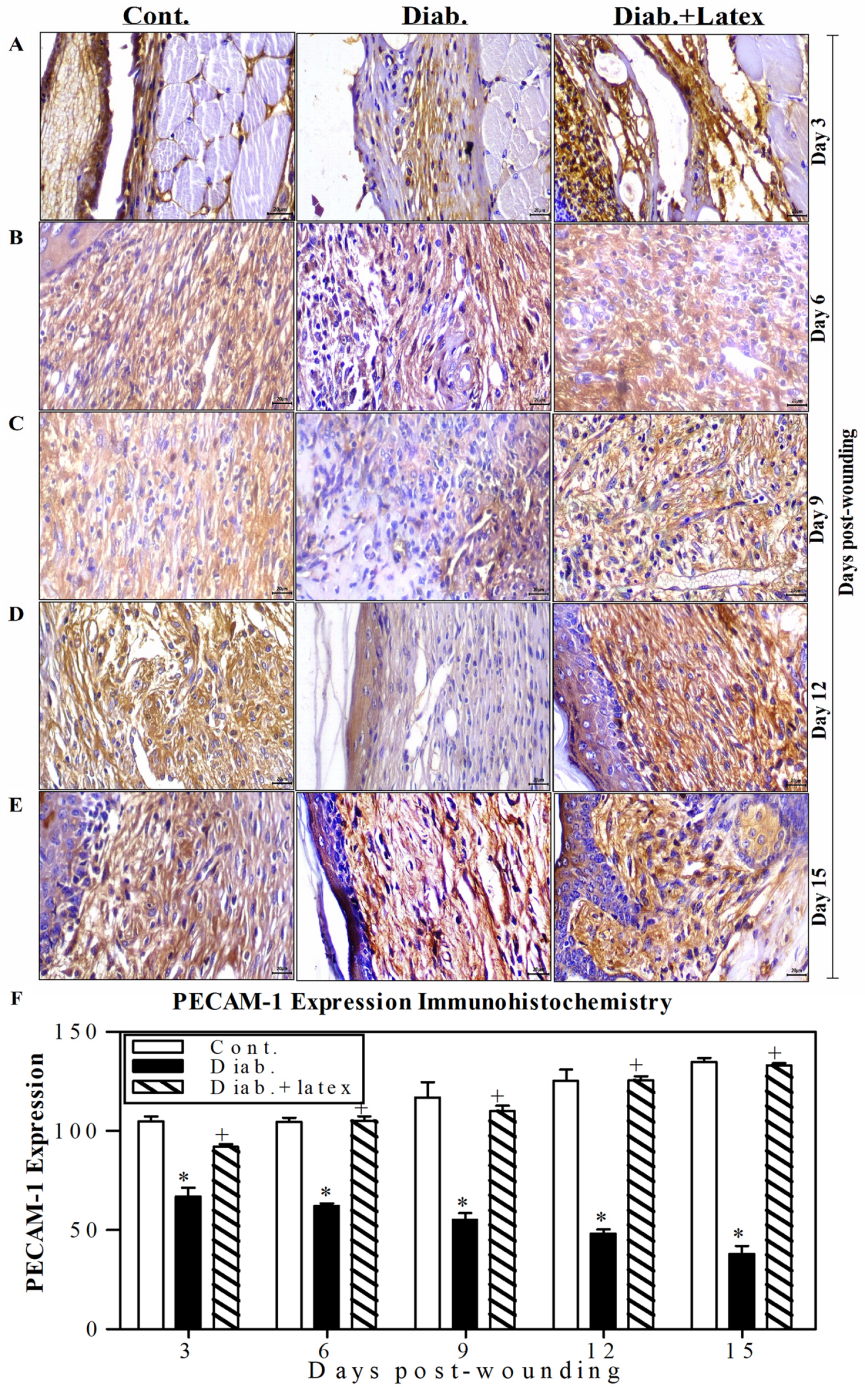
Effects of fig latex on the expression levels of PECAM-1 in wounded diabetic skin tissues. The expression levels of PECAM-1 were detected in the wounded skin tissues of the three animal groups using anti-PECAM-1 and IHC assays. One representative experiment is shown at 3 (**A**), 6 (**B**), 9 (**C**), 12 (**D**) and 15 days (**E**) post-wounding. (**F**) The PECAM-1^+^ cells were quantified in the three animal groups, and the accumulated results from three animals from each group are expressed as the mean percentage of PECAM-1^+^ cells ± SEM in control mice (open bars), diabetic mice (closed black bars) and diabetic mice treated with fig latex (hatched bars). *P < 0.05, diabetic versus control; and ^+^*P* < 0.05, diabetic-treated with fig latex versus diabetic animals.

Figure 8A–E displays representative pictures of the immunohistochemical (IHC) expression of platelet endothelial cell adhesion molecule (PECAM-1) as a marker of vascularization in wounded skin tissues. The diabetic animal group showed a decrease in the expression of PECAM-1 from Day 3 to 15 post-wounding, with a mild positive reaction compared to those found in the control animals, which exhibited a strong positive reaction of PECAM-1 expression. Amazingly, the wounded tissues of diabetic skin mice treated with fig latex exhibited a moderate reaction compared to the control group. Using ImageJ software, accumulated data for PECAM-1 expression were quantified and are presented in Fig. 8F. The diabetic mice showed a significant decrease in the expression levels of PECAM-1 from Day 3 to Day 15 post-wounding. In contrast to the diabetic group, both control and fig latex-treated mice exhibited a significant increase in the expression of PECAM-1 from Day 3 to Day 15 post-wounding.

### Fig latex decreases the expression level of inflammatory CCL2 in wounded diabetic skin tissues

**Figure 9:**
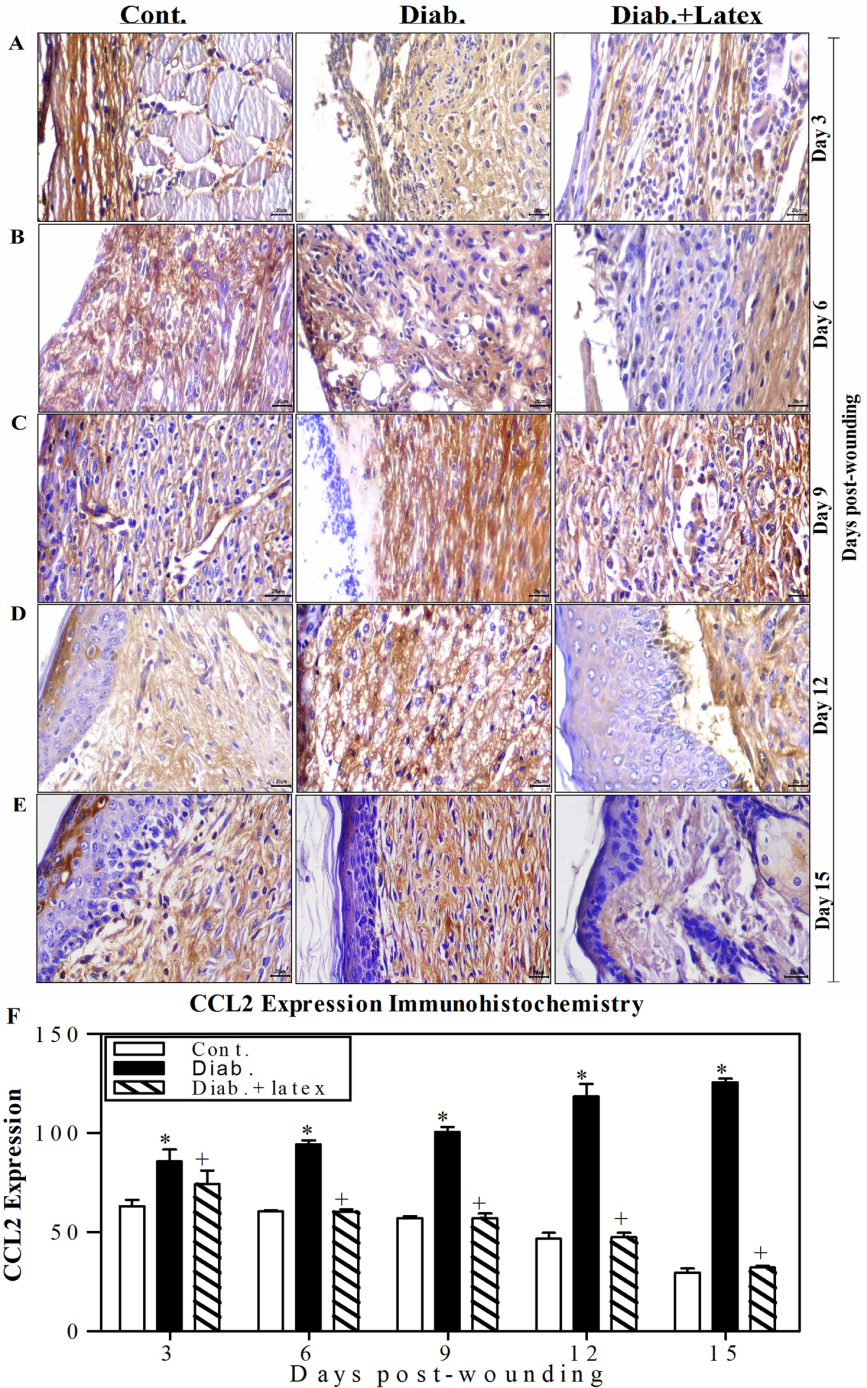
Topical application of fig latex restored the expression levels of CCL2 in wounded diabetic skin tissues. (**A**, **B**, **C**, **D** and **E**) show one representative experiment of CCL2 expression in the control mice, diabetic mice and diabetic mice treated with latex at Days 3, 6, 9, 12 and 15 post-wounding. (**F**) The CCL2^+^ cells were quantified in the three animal groups, and the accumulated results from three animals from each group are expressed as the mean percentage of CCL2^+^ cells ± SEM in control mice (open bars), diabetic mice (closed black bars) and diabetic mice treated with fig latex (hatched bars). *P < 0.05, diabetic versus control; and ^+^*P* < 0.05, diabetic-treated with fig latex versus diabetic animals.

Figure 9A–E shows one representative image of the expression of CCL2 (an inflammatory chemokine). Predominantly, it was noticed that there was increased CCL2 expression in diabetic wounds from Day 3 to 15 after wounding compared to the CCL2 expression in the wounds of the control group, suggesting the presence of the severe complications and protein expression dysregulation associated with diabetes. Topical treatment of diabetic mice with fig latex decreased CCL2 expression to levels similar to those found in the control group. The accumulated data from three animals per group (Fig. 9F) were quantified using ImageJ software. The data demonstrated a gradual significant elevation in CCL2 expression from Days 3 to 15 post-wounding. Nevertheless, the expression levels of CCL2 were significantly decreased in the wounded tissues of the control and fig latex-treated groups with a notable mild reaction.

### Fig latex accelerates the healing process of diabetic wounds via the restoration of the expression levels of β-defensin-1 and ZO-1

**Figure 10:**
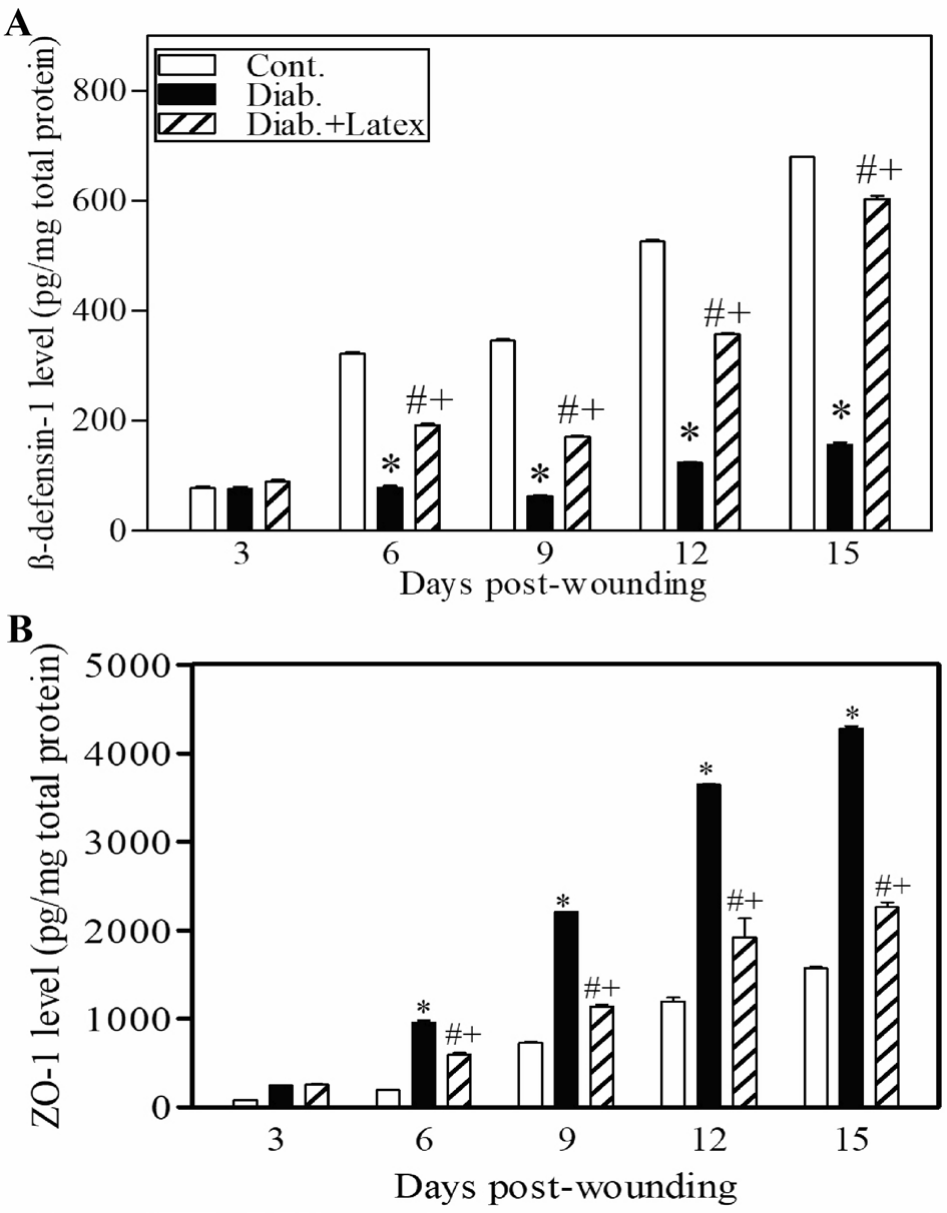
Topical application of fig latex altered the expression of β-defensin-1 and ZO-1. The levels of β-defensin-1 (**A**) and ZO-1 (**B**) in the wounded skin tissues of control mice (open bars), untreated diabetic mice (closed black bars), and diabetic mice treated with fig latex (hatched bars) were measured using ELISA. The collected data from three mice in each group are expressed as the mean ± SEM (n = 3). *P < 0.05 for diab. versus cont.; ^+^*P* < 0.05 for diab. treated with fig latex versus cont.; and ^#^*P* < 0.05 diab. treated with fig latex versus diab. (ANOVA with Tukey’s post-test).

It was concluded that fig latex inhibits pathogenic growth in vitro; hence, the expression levels of an antibacterial protein (β-defensin) and a tight junction protein (ZO-1) were monitored using ELISA and are shown in Fig. 10A, B, respectively. Our results showed decreased levels of β-defensin-1 and elevated levels of ZO-1 in diabetic mice compared to control animals. Interestingly, fig latex-treated diabetic mice exhibited significantly restored levels of β-defensin-1 and ZO-1 compared to vehicle-treated diabetic animals.

## Discussion

Several lines of evidence were used to demonstrate that fig latex exhibited major compounds with powerful biological activities, especially for inhibiting the biofilm formed by the pathogenic bacteria inhabiting diabetic wounds, and their pivotal role for accelerating the healing process. The GC‒MS analysis of fig latex revealed that crude fresh latex contains several phytochemical components, such as terpenes, coumarins, flavonoids and alkaloids^67^. Taraxasterol acetate, which is known as pentacyclic triterpenoid^68^, was only identified in 2006 by Teixeira et al. screening in fig leaves in Portugal^67^. This important compound was detected for the first time and made up more than 50% of the total latex content in fig latex collected in Egypt and has never been recorded before in any previous analysis of fig latex; this may be due to the difference in the geographic distribution. Another important compound is the triterpenoid olean-12-en-3-ol, acetate, also named β-amyrin acetate^69^, which was quantified in the fig latex analysis and represented 32% of the total latex content. Interestingly, some fig latex components identified in GC‒MS analyses have been found to have antimicrobial activities, such as α-gurjunene^70^, nonadecane^71^, germacrenes, pentadecane^72,73^, cis-13-eicosenoic acid, tetracosahexaene^74^, 2,5-di-tert-butyl-p-quinone^75^, cis-vaccenic acid^76^, and lanosta-8,24-dien-3ol, acetate (3.beta. also displayed antifungal and antibacterial activity^77^. Other components, such as humulene^78^, elemene^79^, 1-[5-(2-methylphenoxy)pentyl]piperidine, α-amyrin acetate^80^, and β-amyrin acetate^81^, have shown anti-inflammatory activities, and high-concentration taraxasterol acetate has been shown to have significant antibacterial, anti-inflammatory and anticancer activities^82^. Some of these compounds were identified in our GC‒MS analysis and were detected in several previous GC‒MS analyses of fig latex as previously described^83,84^; these compounds are considered antibacterial phytochemicals. Hence, in the current study, the antibacterial effect of fig latex was due to the fact that more than 83% of the total latex content was taraxasterol acetate and β-amyrin acetate. Most importantly*,* our data revealed the antibacterial activity of fresh fig latex against pathogenic bacteria. Similarly, Aref et al.^84^ proposed that this may be caused by the fact that these two major compounds exhibited similar antibacterial activity against both gram-positive bacteria, especially *Staphylococcus* sp., and gram-negative bacteria^85–87^. Moreover, fig latex was shown to be a therapeutic agent for the complications associated with diabetes, such as an impaired healing process due to the inhibition of insulin sensitivity leading to hyperglycemia^88^, as indicated by the results of the decrease in glucose levels in the mouse model we used after topical treatment with fig latex. In the results of the in vitro study of the antibacterial activity, fresh fig latex showed significant activity against the tested biofilm-forming pathogenic bacteria isolated from diabetic wounds, with MICs of 250–500 mg/ml. This result was also observed in several previous studies, one of them against oral bacteria^89,90^, with MICs ranging from 500 to 750 mg/ml. In addition, the study performed by Aref et al.^91^ investigated the antibacterial effect of fig latex on *Escherichia coli*, *Proteus mirabilis,* and *Staphylococcus aureus* with an MIC of 5 mg/ml. Similar to the results in the study performed in 2014 by Rashid et al., the antibacterial activity of fig latex against *Staphylococcus aureus*, *Streptococcus pyogenes, Escherichia coli, Salmonella typhi, Pseudomonas aeruginosa*, and *Klebsiella pneumoniae* was better than that of gentamycin^92^. Additionally, fresh latex was more effective than stored latex, which confirms the results of previous studies^93^. Our results demonstrated a lack of antibacterial activity of fig latex against the bacterial strain *Pseudomonas* sp. Ms4 (Resistant). The biofilm assay showed that fig latex decreased the biofilm formed by the tested strains isolated from diabetic wounds, especially *Staphylococcus* sp., and it was shown in preclinical trials that fig latex was highly successful for clearing biofilm-forming *Staphylococcus* species^94^. Antibiotics lose their ability to fight bacteria that are protected inside biofilms. In wound treatment, one of the remedies is to boost the antibiotic concentration by a thousand-fold. Another option is to target the biofilm directly, allowing the antibiotic to restore its effectiveness, but all antibiotics used have an adverse effect on health, so the use of fig latex is highly recommended, as it is a natural product that fights pathogenic bacteria. Furthermore, fig latex has the ability to accelerate the wound healing process in the case of diabetic wounds, which may be due its antibacterial activity against the pathogenic bacteria (as shown in the current study) that impaired the healing process^95^. Our data revealed that topical application of fig latex on diabetic wounds accelerated the healing process. Indeed, many factors stall the healing process during diabetes, such as specific metabolic deficiencies, impaired physiological responses and the shrinking of blood vessels^96^. Unexpectedly, topical application of fig latex mediated restoration and increased collagen deposition (as shown by Sirius red staining). Additionally, the expression of PECAM-1 levels was determined in diabetic wounds as a response to the presence of whole bacteria^97^, as shown in Fig. 8. The moderate positive reaction of PECAM-1 was evidenced as a consequence of fig latex activity on the diabetic wounds. The impact of fig latex on wound healing may be due to its ability to decrease the risk of bacteria inhabiting diabetic wounds and increase PECAM-1 expression^98^ because it was previously illustrated that PECAM-1 is necessary for flow-induced vascular remodeling^99^. Interestingly, the treatment of diabetic wounds with fig latex gradually decreased the levels of CCL2 expression and hence inhibited the inflammation of the wounded tissues. In this context, it has been shown that bacterial infection of wounded tissues in diabetic animals leads to a strong increase in the expression of CCL2^100^. These important findings proved the multifunctional ability of fig latex as a good candidate for an anti-inflammatory and antibacterial agent that could accelerate the healing process by downregulating the expression of CCL2. On the other hand, Li et al., 2010 argued that anti-CCL2 antagonists can be used to prevent bacterial infection at the local level^101^. A consequence of β-defensin-1 is constitutive expression in most tissues; it has been suggested as the most significant antibacterial autoimmune defense in living tissues^102^. Here, ELISA analysis demonstrated that the treatment of wounded diabetic skin tissues with fig latex increased the expression levels of β-defensin-1. The antibacterial, anti-inflammatory, and wound-healing abilities of β-defensin-1 promote its use as a prospective strategy to overcome the impaired diabetic wound healing process^103^. As detected in our study, the expression levels of ZO-1 were significantly decreased after the treatment of wounded diabetic skin tissues with fig latex compared to untreated diabetic mice, confirming the upregulation of ZO-1 expression within diabetic epidermal cells, which, in turn, delayed the healing process^104^. In this context, it has been illustrated that the presence of pathogenic bacteria in wounds influences tight junction proteins (TJs), including the ZO-1 protein^105^.

## Conclusions

The impairment of diabetic wound healing was attributed to pathogenic bacteria invading the diabetic wounds and the effects of these bacteria on prolonging the healing phases. Furthermore, disrupted CCL2 and PECAM-1 angiogenic signaling led to impaired and delayed wound healing. Our results illustrate that fig latex accelerated the healing process of diabetic wounds via its direct effect on the survival of pathogenic bacteria and the formed biofilm. Moreover, fig latex improved the healing process by restoring the expression levels of β-defensin-1 and PECAM-1 and collagen formation. Additionally, the treatment of diabetic mice with fig latex regulated the expression levels of ZO-1 and CCL2, suggesting the efficacy of fig latex as a natural candidate for the management of diabetic wounds.

## Material and methods

### Plant latex extraction and storage conditions

The latex of figs was collected from June to August 2019 and 2020 from unripe inedible fig fruit from plants located at the site “N 26°55′51″ E 31°29′17- El Badari-Assiut-Egypt”.

The latex was collected in 1.5 ml Eppendorf tubes and was stored in an icebox during the collection time. No official or specific permits were required for the previously described location or for plant latex collection. The plant used in the present study is not protected or endangered. A milky white liquid (latex) bleeds out of the green unripe fruit when they are being cut. The latex was collected at its peak of activity in the early morning (6–8 am). The latex was obtained by placing a sterile 1.5 ml Eppendorf tube under the cut site of the fruit, collecting approximately 10 ml (1 ml in each Eppendorf tube) and storing the latex by putting these tubes in an icebox to be used on the same day of collection. Latex not used after 12 h of its collection from the tree was discarded because of coagulation^106^. Fig latex was sterilized by filtration using a filter with a pore size of 0.45 μm, collected in a labeled sterile screw-top tube and stored at − 20 °C until use. The plant experiments were performed in accordance with relevant guidelines and regulations.

### Gas chromatography‒mass spectrometry analysis

The analysis of *Ficus carica* latex hexane extract was performed on a gas chromatograph‒mass spectrometer (Agilent Technologies, GC Model 7890A coupled with inert MSD Model 5975B, USA) equipped with a J&W capillary DB-5MS column (30 m in length; 0.25 mm i.e.; 0.25 mm film thickness) and an ionization voltage of 70 eV. The carrier gas was He with a flow rate of 0.5 mL/min to 10.9 min and then ramping to 1 mL/min for 40 min by increasing the rate to 1 mL/min. The oven temperature program was as follows: 40 °C for 2 min, followed by ramping of 10 °C/min to 150 °C for 3 min, and then 220 °C by a flow rate of 10 °C/min for 6 min. Finally, 280 °C was achieved with ramping of 15 °C/min for 20 min. The chromatograph was equipped with a split/splitless injector used in the split mode. The split ratio was 1:100. The control of the GC‒MS system and the data peak processing were carried out using MS Hunter software. The identification of components was assigned by matching their mass spectra with Wiley and NIST library data. Sample analyses were carried out at the Analytical Chemistry Unit (ACAL), accredited by the American Association for Laboratory Accreditation (A2LA), Assiut University, Egypt.

### Patients, bacterial strains and culture conditions

The study was approved by the ethical committee of the Faculty of Medicine, Assiut University, Egypt (IRB no: 17101272). Informed and written consent was given by all participants prior to enrollment. All methods were performed in accordance with the guidelines of the Declaration of Helsinki. The guidelines used for the microbial studies were those of the Clinical and Laboratory Standard Institute (CLSI). Different species of bacteria, including Gram-positive and Gram-negative bacteria, were isolated using the swab method^107^ from diabetic foot ulcers of diabetic patients attending the Diabetes, Endocrine Centre and Vascular Surgery outpatient clinics in Assiut University Hospitals. All isolates were processed, isolated and identified by standard methods^108,109^, including Gram staining, culture, biochemical reactions, VITEK and 16S rRNA sequencing. The isolates used were *Staphylococcus haemolyticus* AUMC b-331, two *Pseudomonas* species, *Bacillus* sp., and *Paenibacillus* species. Bacteria were cultured in nutrient broth (N.B.) (5 g/L peptone, 3 g/L beef extract and 3 g/L NaCl) for 24 h at 37 °C. The optical density (OD) of the cultures was quantified at 600 nm. The cultures were diluted with 0.9% NaCl (normal saline) to bring the OD value to 0.260, which is equivalent to a turbidity of 0.5 McFarland units [10^6^ CFU/mL]^110^. N.B. was utilized to maintain and grow the bacteria for most of the in vitro bacteriological studies. However, to perform the biofilm assay, tryptic soy broth (TSB) medium consisting of 17 g of tryptone, 3 g of soy, 5 g of NaCl, 2.5 g of dipotassium phosphate (K_2_HPO_4_), and 2.5 g of glucose was dissolved in one liter, autoclaved after gentle heating, and used for *Staphylococcus* sp*.*, and Lauri broth (LB) medium was used for *Pseudomonas* species, *Bacillus* sp., and *Paenibacillus* sp. The absorbance for measuring planktonic and biofilm growth was evaluated using a Multiscan Spectrum (THERMO ELECTRON CORPORATION, FINLAND) ELISA reader^94,111^. Muller-Hinton agar medium was used for the detection of antimicrobial activity.

### Antibacterial activity of fig latex using a well diffusion assay

The conventional well diffusion method used as a screening method to determine the antibacterial efficacy of natural products reported early by^112,113^ and according to NCCLS^114^ was employed for the initial assessment of the antibacterial potential of the fig latex extract. The inoculum of each 18–20 h precultured pathogenic bacterial isolate to be tested containing 10^6^ CFU ml^-1^ was spread using a sterile swab moistened with the bacterial suspension on Muller-Hinton agar plates. Subsequently, wells of 8 mm diameter were placed into agar medium, filled with 100 μl of fresh latex, and kept at room temperature for 2 h to allow diffusion^115^. Then, the plates at 36° ± 1 °C for 24 h were incubated under aerobic conditions in an upright position. Standard antibiotic discs of oxacillin (5 μg/disc), tetracycline (30 μg/disc), methicillin (MET, 5 mcg), and cefotaxime (CTX, 30 mcg) were used. All tested antibiotic discs were served as positive reference standards to determine the sensitivity of the bacterial stains tested. The clear zone surrounding the well (microbial growth inhibition zone) was measured as the diameter in millimeters (mm), and three replicates were performed against each of the pathogenic bacteria tested.

### Determination of MIC by the broth macrodilution method and the MBC

Serial twofold dilutions (500, 250, 125, 62.5, 31.25, 15.62, 7.81, 3.9, 1.95, 0.97 mg/mL) were prepared in N.B. in sterile tubes, and their OD values were measured at 600 nm^110^. Then, these tubes were inoculated with 100 μL of bacterial suspension and incubated at 37 °C for 24 h. Using a spectrophotometer with a wavelength of 600 nm, the OD values of each test tube inoculum were measured. These values were subtracted from those obtained prior to incubation. This subtraction is important to exclude the interference in absorbance due to the color of the latex. Inoculated test tubes with zero or near-zero OD values represented the MIC of latex^116^.

### Determination of the MBC

We determined the MBC value; tubes with MIC values were subcultured on freshly prepared sterilized nutrient agar (N.A.) plates. Then, the plates were incubated at 37 °C for 24 h, and the growth of bacteria was observed. A decrease in colony count by 99.9% from the original bacterial inoculum was taken as the MBC value^117^.

### Anti-biofilm assay

We designed a microtiter plate to test eleven isolates from diabetic feet to determine which of them could form biofilms and to determine the ability of latex to inhibit biofilm formation. The quantitative biofilm formation and planktonic growth were measured by the method described by Kolter et al.^118^ and demonstrated by Elamary et al.^119^. The recovered DFU pathogenic bacterial isolates were tested for their biofilm activity in a 96-well microtiter plate according to^119,120^ as follows: Briefly, isolates were grown on tryptic soy agar (TSA) for 48–72 h as indicated under static conditions at 37 °C to obtain rigid biofilm structures^121^. The samples were suspended and diluted in TSB, adjusted to an OD_620_ of 0.06 ≈ 0.5 McFarland standard, which contains approximately 1 to 1.5 × 10^8^ CFU/mL. Then, 130 μl from each isolate culture was plated into a 96-well microtiter plate (U bottom), and 30 μl of fresh latex was added at different concentrations, followed by incubation for 24 h at 37 °C under aerobic and static conditions^119^. Planktonic growth was detected by measuring the absorbance of each well at 600 nm using a microplate reader. Then, the plate was washed three times using 200 μl of sterile PBS to remove the grown culture and the nonadherent cells (washing step). Then, to detect the anti-biofilm activity of fig latex, the wells were air dried; consequently, the adherent biofilm was stained using 200 μl of 0.1% freshly prepared crystal violet for 10 min. The excess stain was removed by rinsing three times with 200 μl of PBS or sterilized distilled water. Furthermore, 100 μl of 70% ethanol was added and incubated for 30 min to stain the crystal violet (solubilization step). Finally, the absorbance of each well was measured at 600 nm using a microplate reader^119^.

### Survival curve of the DFU pathogenic bacterial isolates in the presence of fresh fig latex

The increase in total cell mass and cell number was quantified by estimating the turbidity of a bacterial suspension using a spectrophotometer. Therefore, the suspension at the beginning and completion of the experiment was measured. Isolates were grown overnight on TSA plates, two tubes for each isolate suspended in TSB to an OD600 of 0.01. The first tube of each isolate was considered the positive control, and the other tube was incubated with the MBC value of fig latex. All tubes for each isolate were incubated at 37 °C. An aliquot of approximately 1 ml was tested from the culture medium over time (0, 4, 8, 12, 16, 20, and 24 h) to monitor the value of the optical density of all bacterial treatments at OD600 using the “MIOSTECH, INC. (USA), UV-120 SPECTROPHOTOMETER”. Readings were taken three times. The results were confirmed by taking 50 μl of each treatment at an OD600 of 0.0 (complete killing) onto fresh TSA and incubating at 37 °C for 24 h (three plates were used for each isolate).

### In vivo wound closure studies and ethical issues

In total, 60 healthy BALB/c strain male mice (25–30 g) 4 to 5 months old were procured from the VACSERA animal farm, Helwan—Cairo, Egypt. The animals were housed in standard animal environmental conditions (25–28 °C, 65–70% relative humidity and a 12-h/12-h light/dark cycle) for one week for adaptation. During the experiment, the mice were fed a standard pellet diet and given water ad libitum. The Animal Ethics Committee (AEC) of the Faculty of Medicine, Assiut University, approved the protocol used in this study according to the National Institutes of Health (NIH) guidelines. The study was also reported in accordance with ARRIVE guidelines. We made all efforts to minimize animal distress and reduce the number of animals used in this study. Streptozotocin (STZ) was purchased from Sigma Chemical Co. (St. Louis, MO, USA). STZ was freshly prepared for immediate use (within 5 min) by dissolving in cold 0.01 M citrate buffer (pH 4.50). All mice were fasted for 20 h prior to diabetes induction. The mice (n = 40) were rendered diabetic via daily intraperitoneal (i.p.) injection of STZ (60 mg/kg body weight in 0.01 M citrate buffer, pH 4.5) for 5 consecutive days, and mice in the control group (n = 20) were injected with vehicle alone (0.01 M citrate buffer, pH 4.5). Blood glucose levels were monitored by a glucose meter (BIONEME GM300, TAIWAN) three days after the induction of diabetes. Blood was taken from the tail vein, and glucose levels were determined. Mice with blood glucose levels > 250 mg/dL were considered diabetic mice^122^. The animals were housed for 2 weeks prior to wounding and fig latex treatment to generate a chronic diabetic animal model (glucose level exceeding 250 mg/dl).

### Experimentally inflicted wounds and macroscopic examination

Two weeks following diabetes induction, the mice were wounded as previously described^123,124^. Briefly, the mice were anesthetized with a single i.p. injection of ketamine (80 mg/kg body weight) and xylazine (10 mg/kg body weight)^125,126^. The back of each mouse was shaved and cleaned with 70% ethanol^127,128^. Two wounds (8 mm in diameter) were made on the back of each mouse by excising the skin and underlying panniculus carnosus^129^. Mice were randomly divided into three groups: Group 1, nondiabetic control mice; Group 2, diabetic mice; and Group 3, diabetic mice treated with fig latex. The wounded skin area of mice in the control and diabetic groups was topically treated with 50 μl PBS (vehicle)/wounded area/day for 15 days^128^. However, the wounded area in mice of Group 3 was topically treated with fresh fig latex (50 μl every 12 h/wounded area/day for 15 days). The diameters of wounds were estimated and recorded at the indicated time points as previously described^130^. The optimal dose of fig latex was determined in our laboratory based on the LD_50_ value and several previously established parameters^128^. Skin biopsy specimens were obtained from animals in each group 3, 6, 9, 12, and 15 days after wounding for ELISA and IHC analyses^131^. The diameter of each wound site was measured at the indicated time points to calculate the percentage area of wounds that had closed and healed^125^. Changes in the wound area are expressed as a percentage of the initial wound area. The percentages were calculated using the diameter of steel making the wound (8 mm) as the initial diameter size (D_0_), and the diameter was calculated at every time point under investigation (D_1_): % of wound closure = [(D_1_ − D_0_)/D_1_] × 100.

### ELISA analysis

A 2.0-mm punch biopsy taken from the wound area was collected and frozen in liquid nitrogen. The samples were homogenized in lysis buffer containing protease inhibitors (Roche Diagnostics, Indianapolis, IN), disrupted using Fast Prep (Q-Biogene, Solon, OH), and centrifuged at 5000× *g* for 10 min. The concentration of protein in each lysate was assessed using a bicinchoninic acid (BCA) protein assay kit (Pierce, Rockford, IL), and the supernatants were tested for β-defensin and ZO-1 levels using an ELISA kit (R&D Systems, Lille Cedex, France) according to the manufacturer’s instructions and as previously described^132^. The results were expressed as picograms of target molecule per milligram of total protein for each sample.

### Immunohistochemistry analysis

Samples were isolated from animals 3, 6, 9, 12 and 15 days post-wounding for histopathological examination. The skin specimens were immediately fixed in formal alcohol until processed^133,134^. Each specimen was then dehydrated and embedded, and thin sections (3 μm) were prepared. For immunohistochemistry, tissue sections were processed and stained with the following primary antibodies (anti-PECAM-1 and anti-CCL2) (Santa Cruz Biotechnology).

### Statistical analysis

Statistical analysis was performed based on normally distributed data, which are expressed as the means ± standard errors of the means (SEM), using GraphPad Prism software version 5. The significant differences among the three groups were analyzed using one-way ANOVA followed by Tukey’s posttest.

## Data Availability

All generated data are included in the published manuscript. For any other information, contact the corresponding author (Gamal Badr: badr73@yahoo.com and gamal.badr@aun.edu.eg).
